# A Performance Improvement for Indoor Positioning Systems Using Earth’s Magnetic Field

**DOI:** 10.3390/s23167108

**Published:** 2023-08-11

**Authors:** Sheng-Cheng Yeh, Hsien-Chieh Chiu, Chih-Yang Kao, Chia-Hui Wang

**Affiliations:** 1 Department of Information and Telecommunication Engineering, Ming Chuan University, Taoyuan City 333, Taiwan; peteryeh@mail.mcu.edu.tw; 2 Department of Computer and Communication Engineering, Ming Chuan University, Taoyuan City 333, Taiwan; s871901@yahoo.com.tw; 3 Department of Computer Science and Information Engineering, Ming Chuan University, Taoyuan City 333, Taiwan; wangch@mail.mcu.edu.tw

**Keywords:** earth’s magnetic field, fingerprinting, indoor positioning, KNN, RSSI, rotation matrix

## Abstract

Although most indoor positioning systems use radio waves, such as Wi-Fi, Bluetooth, or RFID, for application in department stores, exhibition halls, stations, and airports, the accuracy of such technology is easily affected by human shadowing and multipath propagation delay. This study combines the earth’s magnetic field strength and Wi-Fi signals to obtain the indoor positioning information with high availability. Wi-Fi signals are first used to identify the user’s area under several kinds of environment partitioning methods. Then, the signal pattern comparison is used for positioning calculations using the strength change in the earth’s magnetic field among the east–west, north–south, and vertical directions at indoor area. Finally, the k-nearest neighbors (KNN) method and fingerprinting algorithm are used to calculate the fine-grained indoor positioning information. The experiment results show that the average positioning error is 0.57 m in 12-area partitioning, which is almost a 90% improvement in relation to that of one area partitioning. This study also considers the positioning error if the device is held at different angles by hand. A rotation matrix is used to convert the magnetic sensor coordinates from a mobile phone related coordinates into the geographic coordinates. The average positioning error is decreased by 68%, compared to the original coordinates in 12-area partitioning with a 30-degree pitch. In the offline procedure, only the northern direction data are used, which is reduced by 75%, to give an average positioning error of 1.38 m. If the number of reference points is collected every 2 m for reducing 50% of the database requirement, the average positioning error is 1.77 m.

## 1. Introduction

Indoor positioning systems, which are often used in department stores, hospitals, and parking lots, allow rapid positioning in an unfamiliar environment and also provide local area information for navigation. There are many methods to produce precise indoor positioning, but most of them require a device to be deployed and set up; therefore, most indoor positioning services become more costly and need regular equipment maintenance.

The most common methods for indoor positioning, including radio frequency identification (RFID), Wi-Fi, and Bluetooth with low energy (BLE), use the radio signal strength between transmitters and receivers to calculate the possible distances from signal sources. The disadvantage of radio signals is that they are susceptible to human body shadowing and multipath propagation delays, so when the signal strength is poor or shadowed, the accuracy of the indoor positioning would be decreased. Using a fixed access point (AP) for mobile phones, the values for wireless signal strength, which is the received signal strength indicator (RSSI), are applied for recent indoor positioning algorithms. RADAR positioning technology was released by Microsoft in 2000 for indoor Wi-Fi positioning [1,2].

It is also possible to perform indoor positioning through RFID systems, which allow the trajectory of moving objects or people to be obtained. Using integrity constraints for data removal is an effective way of improving RFID track detection accuracy, according to B. Fazzinga et al. [3]. The Bayesian inference-based estimation method was used by Zhao et al. [4] to cleanse the uncertain RFID for increasing track tracking accuracy. To handle false negatives in indoor RFID tracking data, A.I. Baba et al. [5] proposed a probabilistic distance-aware graph using the indoor topology and RFID reader position. By comparing raw RFID data with the probabilistic distance-aware graph, failed readings can be filtered and the missing information can be recovered to enhance the tracking data’s accuracy.

The sampling technology has a large impact on detecting the trajectories of indoor moving objects. According to RFID readable ranges, a novel Metropolis Hastings sampler was proposed by B. Fazzinga et al. [6] for area partitioning and it obtains effective RFID numbers and signals in each partition. With the correct sampling data by Metropolis Hastings sampler, the movement between those RFID readers can be immediately identified and the trajectory is also determined. Based on the hidden Markov model (HMM)-based map matching scheme and travel time estimation method, A. Thiagarajan et al. [7] proposed VTrack system for tracking vehicle trajectory in the transportation system. VTrack can tolerate the shadowing of Wi-Fi and cellular signals and still provide the correct trajectory of a moving car.

Using the earth’s magnetic field [8] alone presents the problem of positioning ambiguity. Therefore, our study will not only collect the magnetic field strength for comparison, but also use Wi-Fi RSSI of APs to divide the environment into several areas to improve the accuracy of geomagnetic positioning. To obtain the same accurate positioning results as in the horizontal attitude of the geomagnetic coordinate values and directionality of the sensor device in a non-horizontal state, the magnetic field strength of the device on its own coordinates will be collected in different attitudes, and then the signal processing of rotation matrix could convert that coordinate values to geographic coordinates for absolute positioning calculation results. And whether different sensing elements effect the positioning results is also considered to verify their accuracy, so this research will test different brands of mobile phones with different sensing elements to understand the applicability and positioning accuracy of this research method.

The novelty of this paper is to propose a system architecture with offline and online procedures to enhance indoor positioning by combining information of earth’s magnetic field strength and Wi-Fi RSSI signal strength, where the area partitioning algorithm is also applied in the system. The brief descriptions are as follows: (1). Use Wi-Fi signal strength and APs’ locations to generate four kinds of the area-partitioned methods, which are 2, 4, 6, and 12, to enhance the accuracy of geomagnetic field strength positioning with KNN algorithm. (2). Using the signal processing technology of the rotation matrix, the signal coordinates of the geomagnetic field strength measured by the smartphone can be cost-effectively normalized. Therefore, the smartphone can generate uniform coordinates consistent with the geomagnetic field direction strength in users’ random hand-held postures conveniently. Meanwhile, the indoor positioning accuracy is further improved. (3). Use both online and offline procedures for indoor positioning. In the offline procedure, the signal strength databases of Wi-Fi and the geomagnetic field strength are established, and the indoor positioning results can be obtained immediately in the online procedure.

The remainder of this paper is organized as follows. The related works are described in Section 2. The third section briefly describes the proposed indoor positioning system architecture. The fourth section details the calibration method for geographic coordinates and sensor differences. The fifth section details the experimental environment and the test results with analysis. The conclusion and future work are finally drawn in Section 6.

## 2. Related Works

There are two kinds of methods for RADAR positioning: signal pattern comparison and a mathematical model. These use either an offline procedure or an online procedure. JP Grisales Campeón et al. [9] used support vector regression (SVR), least absolute shrinkage and selection operator (LASSO), kernel ridge regression, k-nearest neighbors (KNN) [10], and other methods to filter abnormal signal strength. The best method is SVR, which gives an average positioning error of 1.24 m. H. Zhao et al. [11] used signal pattern comparison, but this requires more time and cost to collect data. A universal Kriging was used to estimate the interpolation to reduce the number of reference points and the cost. Q. Lu et al. [12] proposed a dynamic positioning method with pedestrian dead reckoning (PDR), which uses Wi-Fi for positioning and then locates the results with PDR. Data fusion reduces the average positioning error and signal pattern comparison gives significantly better accuracy. Previous studies [13,14] use this method because more complete comparison information is obtained, but more time is required for data collection, so different interpolations can be used to achieve similar results. Wi-Fi positioning technology is mature, and various methods can be used to reduce the average positioning error. However, it is difficult to improve the overall accuracy using only Wi-Fi because the effect from the multipath propagation and the human body shadowing could cause the weakening or distortion of the Wi-Fi signals and result in the uncertainty of the indoor positioning accuracy.

The intensity of the earth’s magnetic field is different from microwave signal distribution because the magnetic field strength distribution is not easily affected by shadowing or multipath propagation. Position jumping is a problem when using only the earth’s magnetic field, without a differential algorithm or other reference information. B. Brzozowski [8] and others proposed the use of geomagnetic features for indoor navigation, using known magnetic field maps and visualized vector maps. This allows accurate positioning, recording the trajectory and using the magnet to interfere with the earth’s magnetism to enable a flying drone to record its trajectory and automatically adjust its height to avoid obstacles. To allow continuous positioning and navigation, an unmanned aerial vehicle (UAV) [15,16] is used to record its trajectory. An established magnetic field map is used to estimate the future position and to reduce position drift. D. K. Haryanto et al. [17] compared the positioning performance of GPS, Wi-Fi, and the earth’s magnetic field in public areas and showed that using the earth’s magnetic field to calculate the positioning information can obtain the minimum error result.

S.-C. Yeh et al. [18] used the earth’s magnetic field for indoor positioning and used a KNN algorithm with weighted values for the three-axis geomagnetic components of the sensing results to reduce the positioning error. R. Kang et al. [19] even used an acceleration sensor to calculate the number of steps in the magnetic field indoor positioning but the initial position requires a longer period to obtain, so positioning is not immediate. Moreover, [19] proposed a rotation matrix to transpose sensor related coordinate into a geographic coordinate system for minimizing the positioning error. Ning, F.-S et al. [20] used particle filters and magnetic field maps to optimize the PDR algorithm to reduce the cost of establishment, increase positioning accuracy, and reduce the cumulative error. Two other studies [21,22] use Wi-Fi, the earth’s magnetic field, and PDR with a Kalman filter to reduce the cumulative error for the PDR and estimate the user’s next position. Z.-A. Deng et al. [21] use the technique of Landmark to reset the estimated positioning data of PDR to obtain the current landmark location before the initial position is known, so the user must obtain the environment information before indoor positioning.

This study combines the methods of related works to design an indoor positioning system with online and offline procedure for easily implementation. In Smartphones, besides Wi-Fi signal, there are many sensors equipped, such as magnetic sensors or acceleration sensors, to collect the data of the environment and the movement of the phones. Therefore, those sensors are usually used to detect the distance, position, or attitude. If these two sensors can be used for indoor positioning, there is no need to add additional sensing elements, which can make indoor positioning more convenient. Although using the earth’s magnetic field strength for positioning is prone to interference by permanent magnets, ferrous materials, and devices that produce an electromagnetic effect, the detected values are usually stable and not changed in the same position. Any method of positioning that uses the earth’s magnetic field alone requires an initial position to increase positioning accuracy. For increasing the indoor positioning accuracy from magnetic field strength, our study uses the RSSI to divide the environment into a different number of areas to experiment the indoor positioning errors. The offline procedure is applied to build the position database for decreasing the time of the online indoor positioning. The device related coordinates of the collected magnetic field strength data are also converted into geographic coordinates by rotation matrix to enhance the accuracy of the indoor positioning results. Different sensing values from different brands of magnetic field sensors are calibrated using the proposed algorithm to allow the system to be used on most smartphones. The table for summarizing the difference between proposed work and related ones is as Table 1.

## 3. System Architecture

### 3.1. Combination of RSSI and Magnetic Field Strength

**Figure 1:**
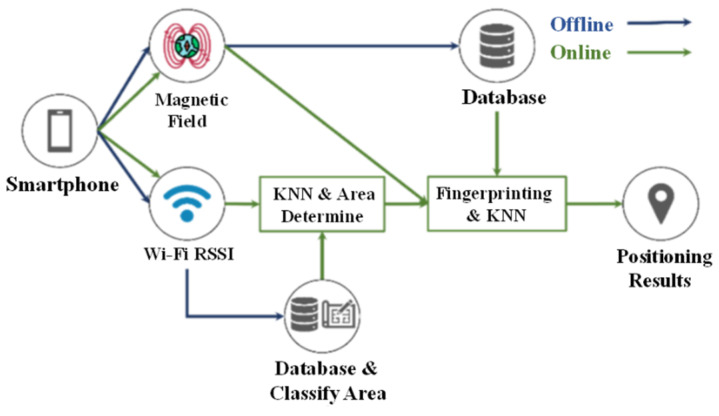
Proposed system architecture.

Figure 1 shows a schematic diagram of the system architecture for this study. The overall architecture uses magnetic field sensors and Wi-Fi modules in smartphones. The system involves offline and online procedures. The offline procedure records all reference points and the magnetic sensor in the smartphone is used to detect the earth’s magnetic field. The Wi-Fi module measures the RSSI of the AP and records four directions at each point, where the distance between two points is designed as one meter. All data are then exported into the magnetic field strength database and the RSSI database for calculating and comparing in the online procedure.

The Wi-Fi data are used to determine the characteristics of the environment and classify the partition results. The collected magnetic field data are then pre-processed to allow averaging. In the online procedure, the phone begins the positioning process, and the system receives the magnetic field data and Wi-Fi data for the specific actual location. The RSSI for the current AP is filtered using KNN to eliminate abnormal signals and is then used to identify the area. KNN modeling does not include a training period as the data represent a model which will be the reference for future prediction and due to this, it is very time efficient in terms of improvising for random modeling on the available data [10]. It is also very easy to implement as the only thing to be calculated is the distance between different points based on data of different features and this distance can easily be calculated using distance formulas such as Euclidian or Manhattan. To make the proposed indoor positioning system process easy to implement and experiment with, the KNN algorithm is applied in this paper. To obtain better position result, the geomagnetism and KNN algorithm are used further to respectively perform the fingerprinting and the data features’ comparison for identified area.

### 3.2. Offline Procedure

**Figure 2:**
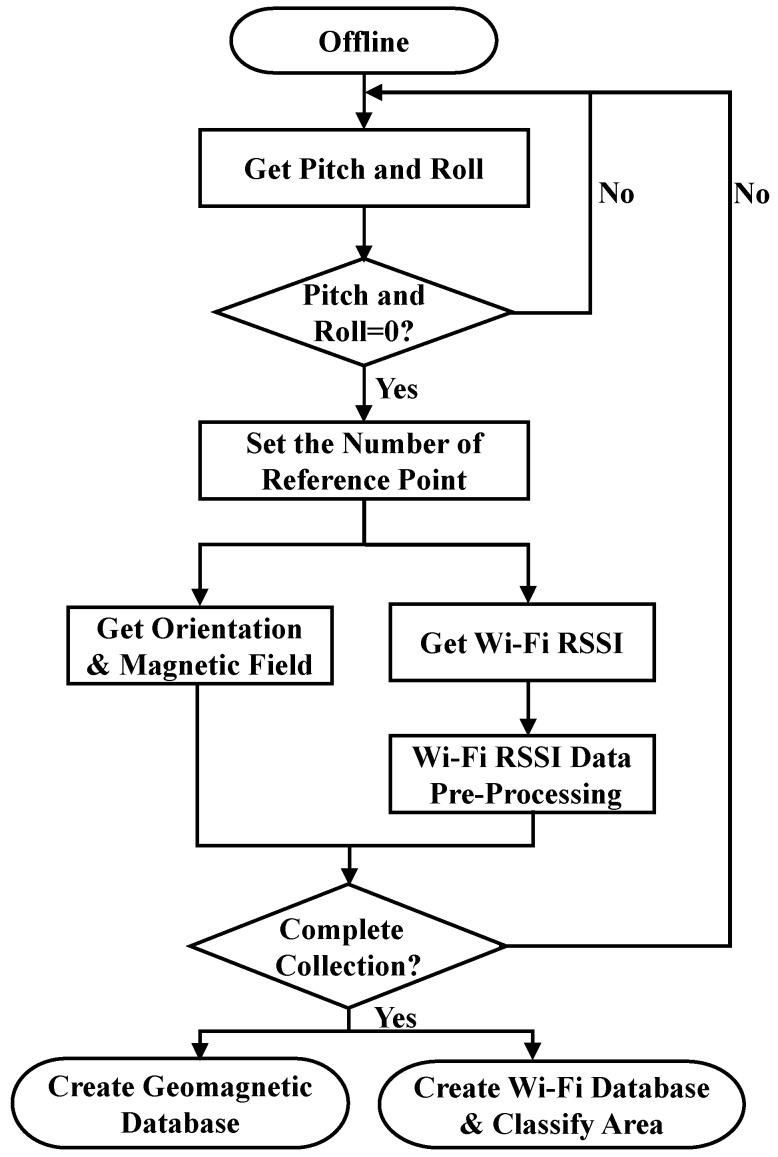
Flow chart for the offline procedure.

Figure 2 shows a flow chart of the offline procedure of this study. The data for each reference point are collected and the mobile phone sensor monitors whether the mobile phone’s pitch and roll angles are 0 degrees. When all sensing values are reset to 0 degrees, the data for the current reference point can be received; otherwise, the system performs the orientation check again.

The azimuth and the reference points that are set by the user are recorded and the geomagnetic component coordinates *x*, *y*, and *z*, are detected. The actual azimuth angle and the azimuth angle that is defined by the recorder are both recorded, including east, west, south, and north data. Then both data are automatically saved into a geomagnetic database and a RSSI database. The RSSI data are also used to divide different numbers of areas, including 1, 4, 6, and 12 areas, in an experimental environment for initial positioning information. There are *m* azimuths (*s* = 0, …, *m*) and *n* common reference points (*i* = 1, …, *n*). The geomagnetic database records the three-axis magnetic field coordinates (*x_i_*, *y_i_*, *z_i_*). The magnetic field strength database matrix **F***_s_* for the *s*th azimuth is shown in Equation (1).

(1) $$F_{s}=\left[\begin{array}{ccc} x_{1} & y_{1} & z_{1}\\ x_{2} & y_{2} & z_{2}\\ \vdots & \vdots & \vdots \\ x_{n} & y_{n} & z_{n} \end{array}\right]$$

The averaging values of the RSSI from *Q* APs are also measured in the offline procedure in *P* pre-selected locations. The RSSI value database matrix **I** generated during offline procedure is shown in Equation (2) where *I_pq_* is the measured RSSI value of the *q*th AP (*q* = 1, …, *Q*) in the *p*th test location (*p* = 1, …, *P*).

(2) $$I=\left[\begin{array}{cccc} I_{11} & I_{12} & \ldots & I_{1Q}\\ I_{21} & I_{22} & & I_{2Q}\\ \vdots & & \ddots & \vdots \\ I_{P1} & \ldots & I_{P-1,Q} & I_{PQ} \end{array}\right]$$

### 3.3. Online Procedure

**Figure 3:**
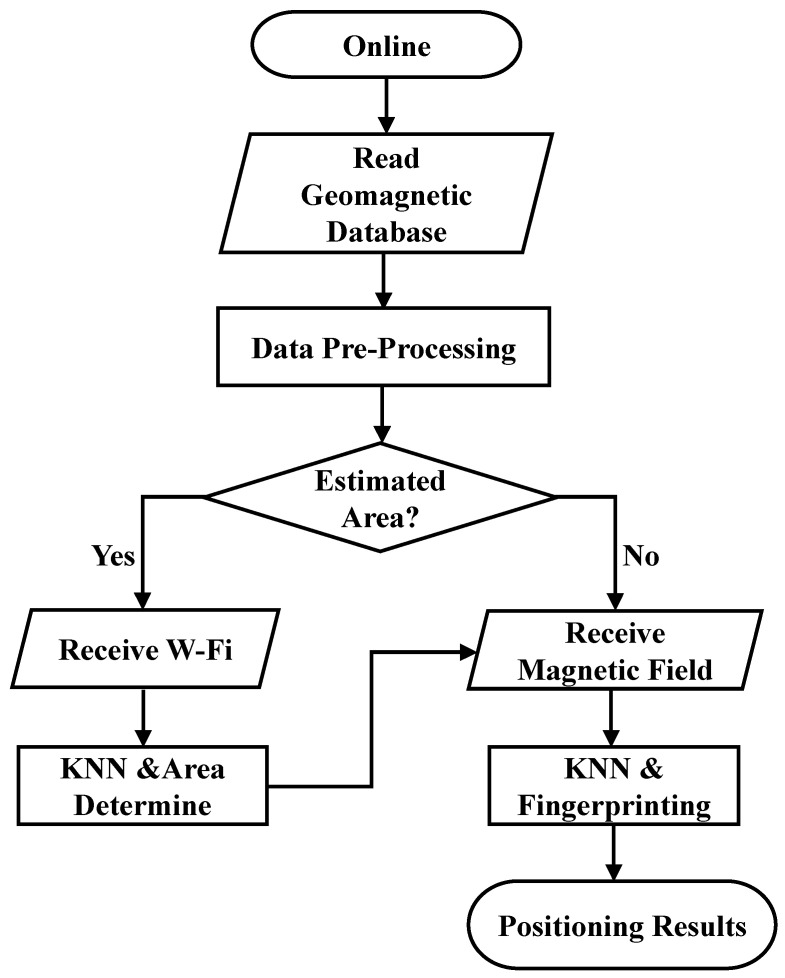
Flow chart for the online procedure.

The online procedure is shown in Figure 3 for calculating the indoor position information on a phone. After reading the magnetic field strength data from the database, the phone uses the measured RSSI values to determine which divided area is to be the initial position information. When the phone continuously receives and measures the RSSI values for several APs at the current location, it passes this information to KNN during the first stage of online procedure. Then KNN uses this information to eliminate extreme signals if the RSSI is too large or the signal is too weak. Meanwhile, the measured magnetic field strength data are used with a fingerprinting algorithm to generate all difference values from magnetic field strength database and then the minimum value will be found for indoor positioning information. The detail calculation of the fingerprinting algorithm for this study is shown in Section 4. The difference values are small when the assumed divided area is correct, or the difference values are large. In the second stage of online procedure, KNN is then used to determine the precise position to obtain the best positioning result.

### 3.4. Area Partitioning Methods

**Figure 4:**
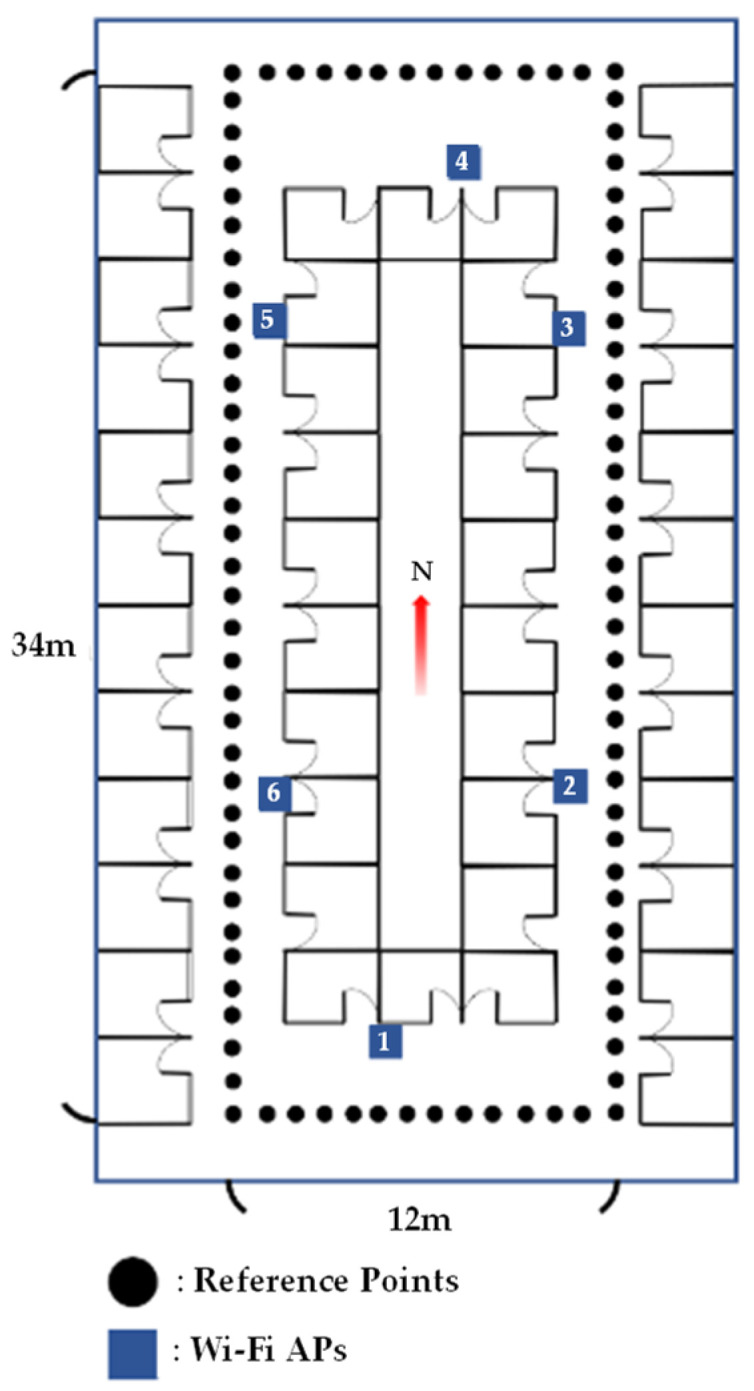
Experimental environment.

Area partitioning could increase the indoor positioning accuracy and prevent the huge positioning error. Area partitioning for proposed indoor positioning procedure is applied in the test environment as shown in Figure 4. The area is 34 × 12 m^2^ and there are 94 pre-defined reference points with one-meter-apart dot points. The blue squares with number 1 to 6 are the locations of the APs. The direction north on the map is marked as N with red arrow. The area partitioning for initial position information is based on the location of the APs and the RSSI measured by testing phone. Moreover, the RSSI values in four directions are also measured and averaged for 94 reference points from testing phones. This study provides four kinds of the area partitioning methods, including 1, 4, 6, and 12.

**Figure 5:**
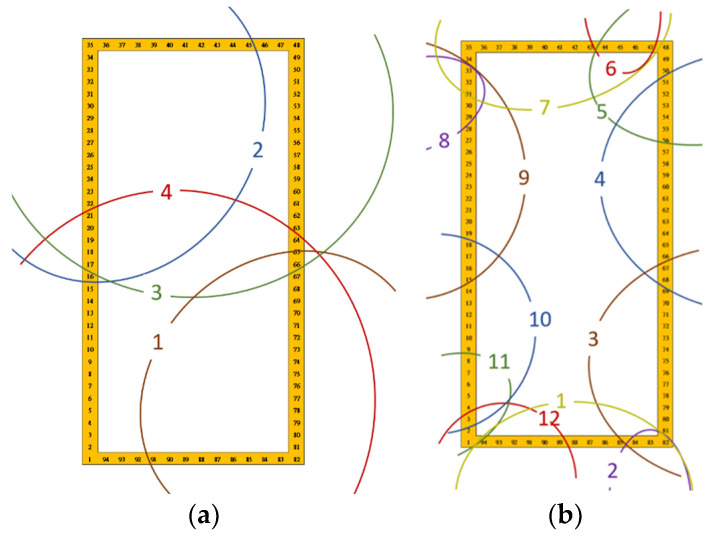
The environment dividing results: (**a**) 4-area dividing results, (**b**) 12-area dividing results.

The 4-area partitioning uses four APs, including AP1, AP3, AP4, and AP6, shown in Figure 5a. The four areas into which the collected APs are classified if there is a received signal, regardless of the strength. If signal coverage areas overlap, the reference point can receive two or more APs so the classification is made using the result for two measured values. This occurs in different directions for the same reference point and the information that is collected is different or less. To reduce the need to identify reference points, 6- and 12-area partitioning are used. When using 6 APs for 12 partitions, the two strongest RSSI signals are measured and recorded at each AP position and the 12-area dividing result is shown in Figure 5b.

### 3.5. Rotation Matrix for Geographic Coordinates

For increasing indoor positioning accuracy, if a handheld mobile phone has a nonzero pitch and roll angle, a rotation matrix [23] is used to transfer the magnetic field strength from device related coordinates into geographic coordinates. This method allows the data for all reference points to be stored in the same database and is not affected by the attitudes of the handheld phone. For this reason, the angles of pitch, roll, and yaw are also measured and record. The rotation matrix in three dimensions is defined by the Euler Angle [24] and Equations (3)–(6) give the geographic coordinates,

(3) $$R_{x}=\left[\begin{array}{ccc} 1 & 0 & 0\\ 0 & \cos\alpha & -\sin\alpha \\ 0 & \sin\alpha & \cos\alpha \end{array}\right],$$

(4) $$R_{y}=\left[\begin{array}{ccc} \cos\beta & 0 & \sin\beta \\ 0 & 1 & 0\\ -\sin\beta & 0 & \cos\beta \end{array}\right],$$

(5) $$R_{z}=\left[\begin{array}{ccc} \cos\gamma & -\sin\gamma & 0\\ \sin\gamma & \cos\gamma & 0\\ 0 & 0 & 1 \end{array}\right],$$

(6) $$\left[\begin{array}{c} X_{o}\\ Y_{o}\\ Z_{o} \end{array}\right]={\left(R_{z}R_{y}R_{x}\right)}^{-1}\left[\begin{array}{c} X_{C}\\ Y_{C}\\ Z_{C} \end{array}\right],$$

where the angle of the rotational pitch **R***_x_* is *α*, the angle of the rotational roll **R***_y_* is *β*, and the angle of the rotational yaw **R***_z_* is *γ*. *X_g_*, *Y_g_*, and *Z_g_* are the geographic coordinates; *X_d_*, *Y_d_*, and *Z_d_* are the coordinate information from the phone [25]. The rotational matrix for this study is applied from Android Studio with function name getRotationMatrix() [26].

### 3.6. Sensing Differences in Mobile Phones

There are many brands of mobile phones and the systems and specifications for each mobile phone differ. If different brands of mobile phones are used to measure the magnetic field strength, the difference in the sensitivity of these mobile phones affects the positioning corrections for the database. This study compares the difference between the device-related coordinates and the converted geographic coordinates for each mobile phone to show whether the conversion of the coordinates increases accuracy. Five different brands are selected and the systems are from Android 7 to Android 11. Magnetometers are supplied by three companies, as shown in Table 2. After testing magnetometers of all phones from AKM, the magnetic field strength data are stable and almost the same in one location. We choose one of the phones using its magnetometer from AKM, which is Mi Lite8, for building the database of the offline procedure. In the online procedure, all phones with different magnetometers in Table 1 are compared for indoor positioning.

## 4. Experimental Environment and Test Procedures

To measure changes in the magnetic field and the RSSI, this study uses the API that is provided by the Android Studio 4.1.2 development platform for the experimental environment. Using the Wi-Fi module to collect the current known number of APs and RSSI allows the tester to obtain a sampling data per 1 msec for at least 10 values. The customized APP for collecting Wi-Fi RSSI and earth’s magnetic field strength values is generated to store all measuring values from 94 selected locations in the experiment environment as Figure 4. The android functions wifi.getScanResults() and level() are used to obtain the RSSI values. The magnetic field strength values are collected by android function sensorManager.getDefaultSensor(Sensor.TYPE_MAGNETIC_FIELD). All five smartphones operate and collect data simultaneously in the same location.

### 4.1. RSSI Measurement and Online Calculation

**Figure 6:**
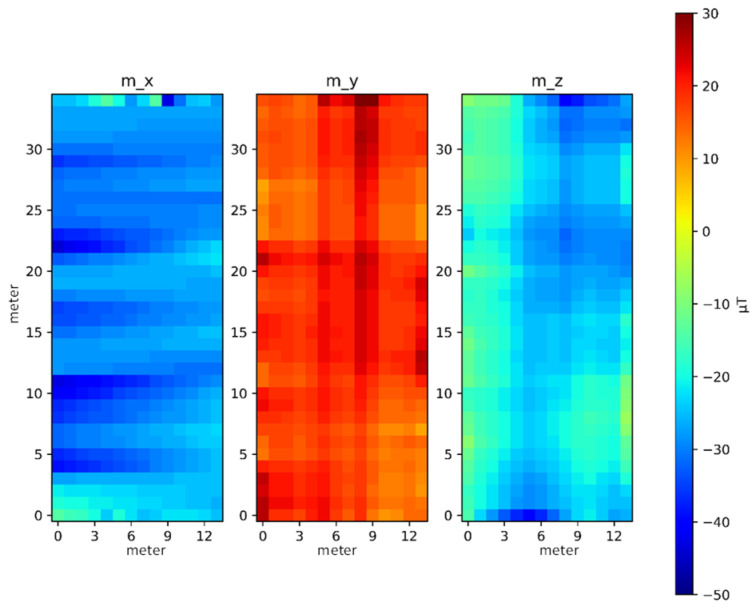
Hot spot distribution for triaxial magnetic field intensity.

Figure 6 shows the heat map for the three-axis (*x*, *y*, and *z*-axis) magnetic field strength for the north magnetic field for the face of this environment, where the horizontal and vertical directions of the experimental environment are the x and y axes. All reference points are measured, which is equivalent to a circle outside. 

**Figure 7:**
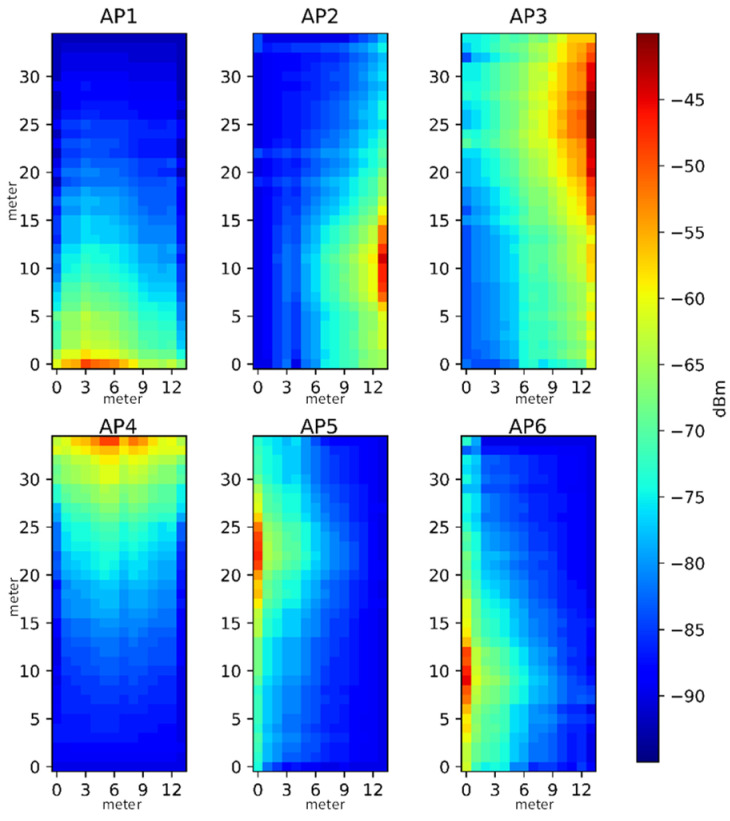
Hot spot distribution for each AP signal intensity.

Figure 7 shows the RSSI heat map for each AP, where the horizontal and vertical coordinates are the x and y coordinates of the experimental environment. The distribution of AP1 to AP6 is shown by the numbers in the blue squares in Figure 7, where the largest strength for each AP is in at least one corridor.

The offline procedure collects the reference point. The data type is APs (RSSI_1_, RSSI_2_, RSSI_3_, …, RSSI_10_) and the geomagnetic and Wi-Fi databases are established together to allow the collected data to be recorded at the same location. There are four pre-defined directions and 94 reference locations. In every reference location, the signal strength will be sampled 10 times per 1 msec. After averaging the measured 10 data, the average signal strength data for the six APs will be used for area partitioning. When connecting with one AP to collect the RSSI signal strength from all APs, the MAC address of connecting AP is also record for different area partitioning methods.

The online procedure uses RSSI to measure the signals, so it is very slow and fewer data are collected than are collected offline. To decrease the collection time, this study uses KNN to eliminate anomalous signal intensities with *k* = 3, which could keep less time in collecting RSSI, so the results for three collections are used. A fingerprinting algorithm could help to find the correct area in Equation (7) by calculating the difference between RSSI in the database from the offline procedure and measured RSSI in the online procedure.

(7) $$d_{pq}=\sqrt{\sum_{i=1}^{n}{\left(r_{i}-I_{pq}\right)}^{2}}$$

where *r_i_* is measured RSSI in the online procedure and *I_pq_* is the RSSI in the database from the offline procedure. The minimum value *d_x_* of the calculated distances *d_qp_* from fingerprinting algorithm is shown Equation (8). Then, the accurate area can be found in the online procedure.

(8) $$d_{x}=\min\underset{pq}{\arg}\;d_{pq}$$

### 4.2. Magnetic Field Strength Measurement and Online Calculation

**Figure 8:**
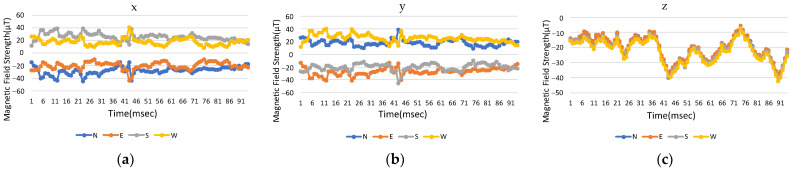
Original measured magnetic field strength in three-axis coordinates with time: (**a**) *x*-axis, (**b**) *y*-axis, and (**c**) *z*-axis.

**Figure 9:**
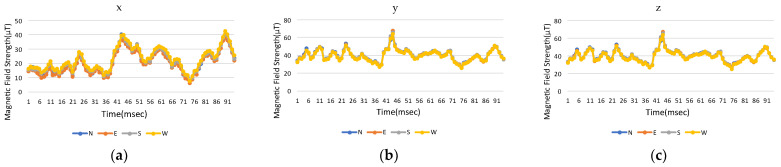
Modified magnetic field strength in three-axis coordinates with time: (**a**) *x*-axis, (**b**) *y*-axis, and (**c**) *z*-axis.

The same mobile phone is initially used to measure changes in the earth’s magnetic field in the experimental environment and the *x*, *y*, and *z* for four directions for the reference point are measured. The measured magnetic field strength of smartphones at 0 degree of pitch and roll, where smartphones are held horizontally with the screen facing up, are shown in Figure 8a–c for the three-axis coordinates in different directions. The changes in the magnetic field strength in the *x* and *y* directions are different and there is a difference in positive and negative values, but the *z*-axis value is almost unchanged. When the absolute value is added, the magnetic field strengths for the three-axis coordinates are shown in Figure 9a–c), where the *x*, *y*, and *z* three-axis curves in different directions are almost the same, but this method also removes the directionality and minimize calculation effort.

For the offline procedure, data are collected at each reference point. The data type is *M_siC_*(*x_i_*, *y_i_*, *z_i_*). In total, 94 reference points are placed and each reference point has four custom directions. The mobile phone has a pitch and roll angle of 0 degrees before recording. For the online procedure to measure the magnetic field strength, the geomagnetic database is read and the data in each reference point are averaged. The final magnetic strength data type is ${\bar{M}}_{si}\left({\bar{x}}_{i},{\bar{y}}_{i},{\bar{z}}_{i}\right)$.

During the online procedure, the kth measured magnetic field strength values (*x_k_*′, *y_k_*′, *z_k_*′) in the current position are from the sensor of the phone. The system compares (*x_k_*′, *y_k_*′, *z_k_*′) with ${\bar{M}}_{si}\left({\bar{x}}_{i},{\bar{y}}_{i},{\bar{z}}_{i}\right)$ in the magnetic field strength database. According to fingerprinting algorithm, the difference values *P_i_* between (*x_k_*′, *y_k_*′, *z_k_*′) and ${\bar{M}}_{si}\left({\bar{x}}_{i},{\bar{y}}_{i},{\bar{z}}_{i}\right)$ is as Equation (9). And the minimum value of *P_k_* is determined in Equation (10).

(9) $$P_{ki}=\sqrt{{\left({x^{\prime }}_{k}-{\bar{x}}_{i}\right)}^{2}+{\left({y^{\prime }}_{k}-{\bar{y}}_{i}\right)}^{2}+{\left({z^{\prime }}_{k}-{\bar{z}}_{i}\right)}^{2}}$$

(10) $$P_{k}=\;\min\underset{i}{\arg}\;P_{ki}$$

The, KNN algorithm is used to minimize the error *P_k_*, where *k* = 3 gives the best result [23]. The phone sensors for this study quickly collect the three results and KNN is used to obtain the final positioning result. 

## 5. Experimental Results

### 5.1. Positioning Errors under Different Area-Dividing Methods

The average positioning errors for geomagnetic positioning are shown in Table 3, for an experimental area that is partitioned into 1, 4, 6, and 12 areas. For increasing the number of the dividing areas from 1 to 12 areas, the average positioning error is decreased by 90%.

**Figure 10:**
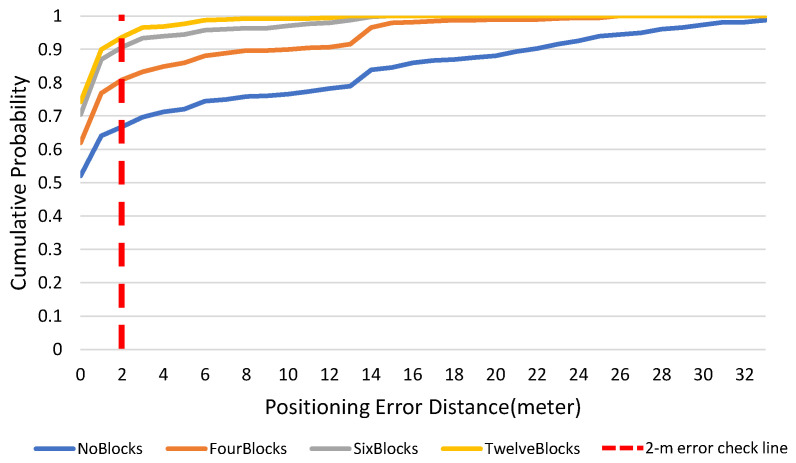
Cumulative distribution function for positioning error by partition type.

Figure 10 shows the cumulative distribution function (CDF) of position errors by p. This determines the probability that the user can accumulate within a certain error range. The results in the figure show that the cumulative probability of occurrence within a 2-m error is at least 80% in the partitioned state, which is 27% more accurate than the result for an un-partitioned state.

Following manual identification of the number of area partitioning and the optimized weighting values for the three-axis magnetic field strength, the average positioning error of EMF [18] and the proposed method without coordinate conversion by rotation matrix are shown in Table 4. Under the optimal weighting values for three-axis magnetic field strength, the EMF method divides the area into four with 2 m average positioning error, which is 0.18 m better than the proposed method. Although EMF has a below-average positioning error than the proposed method in 4-area partitioning, the experiment area of the proposed method is larger than EMF. Furthermore, when the number or the range of areas changes, the EMF method has a more complex procedure than the proposed method. This is because EMF has to manually recognize the changes in the number of partitioning areas by users and the proposed method can automatically update and recognize the changes. Therefore, average positioning error of 12 areas is not achieved in EMF but 12-area partitioning could be operated in this study, which resulted in a small average positioning error of 0.57 m.

After converting the coordinates by the rotation matrix, Table 5 shows the average positioning errors for different numbers of partitioning areas where the weighting values for the three-axis magnetic field and the number of the area partitioning are automatically set in this work. Through the complete procedure of this work, the 4-area partitioning average positioning error is 1.56 m, which is better than that of the EMF method in the 4-area partitioning case. The average positioning error for the new coordinates using the rotation matrix is 1.36 m in the 12-area partitioning experiment.

Table 6 shows the average positioning error for different pitches, with or without conversion coordinates for 12 areas. For a pitch of 0 degrees, 30 degrees, and 60 degrees, the original coordinates are affected by the angle and the original positioning error is 3.41 m. The conversion coordinates are only slightly affected, and the positioning error is less than 1.4 m, which is 68% improvement in 30 degrees pitch angle.

**Figure 11:**
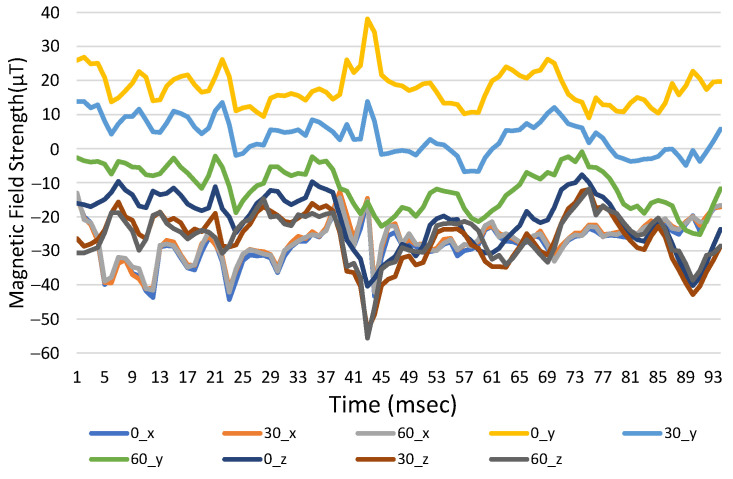
Three-axis magnetic field strength with time for initial coordinates at different angles.

**Figure 12:**
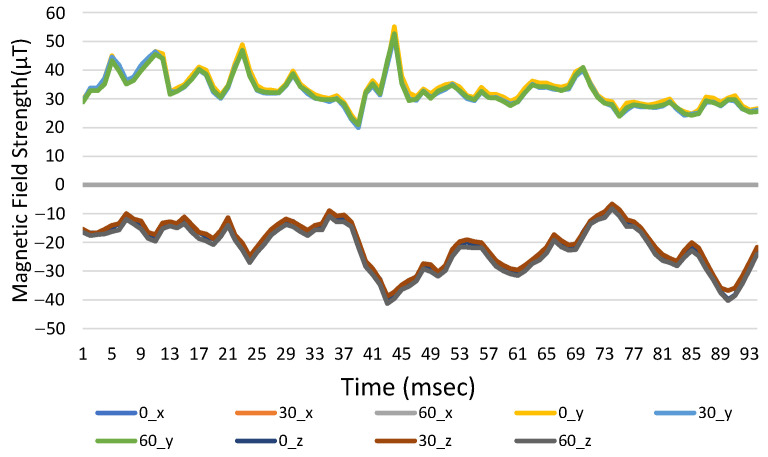
The three-axis magnetic field strength with time for the converted coordinates at different angles.

Reference points and three-axis magnetic field strength for different orientation angles are shown in Figure 11, where the direction is to the north. If the pitch angle in the *x*- and *y*-axis changes, the intensity for the original coordinates changes significantly. When the coordinates are converted, there is no difference between each axis for several pitch angles, as shown in Figure 12.

### 5.2. Calibration Results for Different Brands of Magnetic Field Sensors

**Figure 13:**
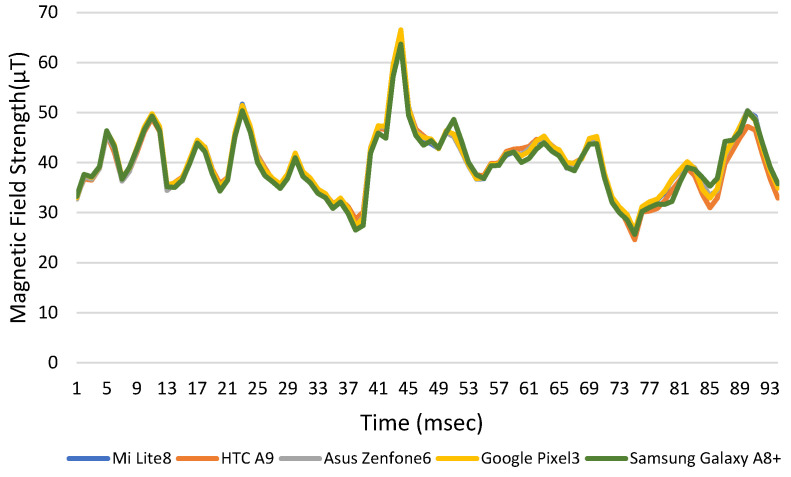
Magnetic field strength with times for different brands of mobile phone.

The average positioning errors for the different brands of smartphones are similar if using different magnetometers in the proposed method. The values of the magnetic field strength are measured similarly, as shown in Figure 13. Table 7 shows the average positioning errors for different smartphones when the original coordinates are classified into 12 areas with angle of 0 degree. The positioning error for all mobile phones is 0.41 to 1.15 m less than 1.2 m. Therefore, the initial coordinates do not affect very much in the overall positioning accuracy.

**Figure 14:**
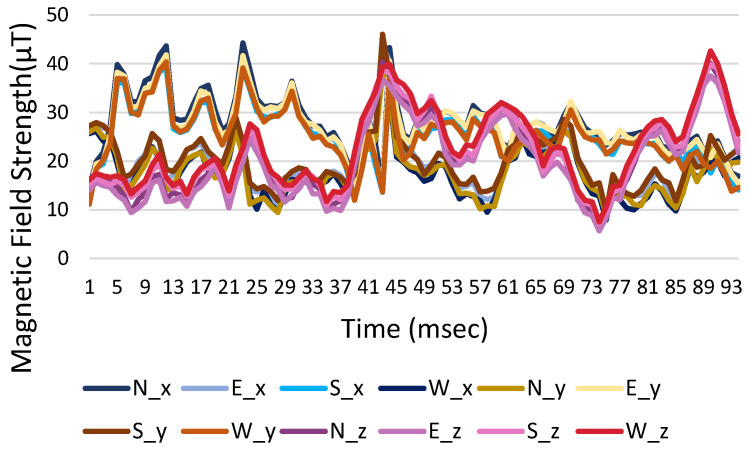
Three-axis magnetic field strength with time using the original coordinate database.

Using 12 areas in the converted coordinates for different mobile phones with pitched angles of 0 degree, the average positioning errors are shown in Table 8, where the average positioning error is 0.80 to 1.96 m. Although the positioning error obtained by most of the phones by converting the coordinates is relatively large, the error values are all very small, and even a phone Google Pixel3 can obtain a smaller positioning error than in the original coordinates. Furthermore, if the pitched angles are less than 60 degrees, the positioning errors for different brand mobile phones could remain almost the same with pitched angle of 0 degree. In Figure 14, all the values are added to the absolute value without direction information. It shows that there are similar values for the coordinates of the three axes at four different azimuth angles. For reducing the amount of data in the database, we can only use the north data and add the absolute value to the geomagnetic database. However, positioning errors could be introduced as well.

**Figure 15:**
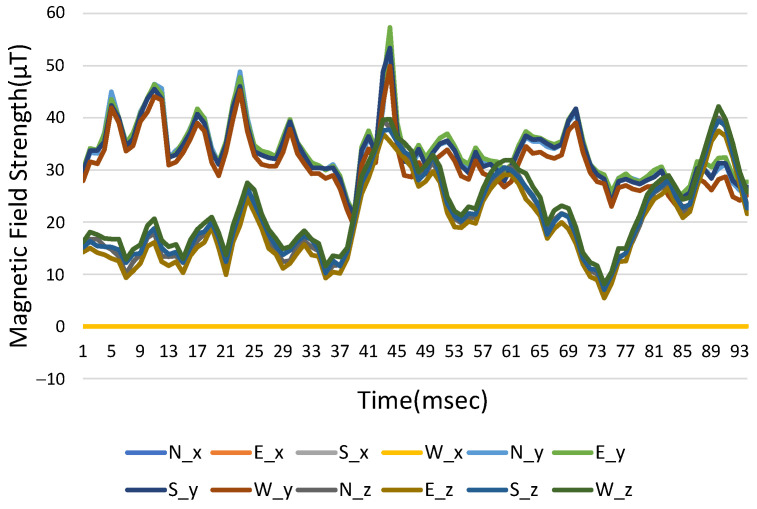
Converting the three-axis magnetic field strength with time in each direction of the coordinate database.

Based on absolute values in Figure 14, Equation (6), and getRotationMatrix(), we can calculate the overall average positioning error as 1.30 m, just slightly increasing by 0.57 m. According to the database of the converted coordinates in Figure 14, if only the northern data, which are reduced by 75% for positioning, are used for the offline database in 12-areas partitioning, an average positioning error of 1.38 m can be calculated from Figure 15, Equation (6), and getRotationMatrix(). In Figure 15, the magnetic field strength in the west direction of *x*-axis (W_x) remains at 0 μT because the rotation matrix converts and rotates the coordinates in this direction.

### 5.3. Efficiency Discussion and Experiment Results

For decreasing the calculation time and the data capacity of the database, we try to use fewer measuring reference points’ data from the offline procedure by increasing distance between each reference point. We even just use only the northern direction offline data to process the indoor positioning through the proposed method. The average positioning errors using all azimuth databases for different distances between reference points are shown in Table 9. If the original coordinate is 1 m, the positioning error is 0.57 m. For distances of APs at 2 m or 3 m, the error respectively increases to 1.28 m and 1.33 m and the error for the converted coordinates increases from the original 1.36 m to 1.57 m and 1.88 m. 

If the azimuth database is reduced, in which only the northern direction database is used, and distances between each reference point are extended from 1 m to 2 m and 3 m, the experiment results of indoor positioning errors are shown in Table 10. Under original coordinates, the error increases from 0.57 m to 1.5 m and 2.94 m. If using coordinates conversion, the error increases from the original 1.36 m to 1.77 m and 2.89 m. Whether extending the distance between reference points or using less azimuth database, the proposed indoor positioning algorithm could provide a positioning error of less than 3 m. Although the average positioning errors in the converted coordinates are larger than that in the original coordinates, almost the same results could be obtained in the different pitched angles of the mobile phones in the converted coordinated.

## 6. Conclusions

In this study, measurement of the magnetic field strength and Wi-Fi signal use built-in sensors in smartphones and no additional sensor or instrument is needed to obtain the indoor positioning information. Wi-Fi signal is used to partition an environment and the signal strength is first filtered using KNN to eliminate abnormal values. Signal pattern comparison is used to compare the characteristics of the earth’s magnetic field. KNN is used to determine the precise location and the result is optimized for 12 areas. The indoor positioning of 6-area partitioning is simpler than that of 12-area partitioning, but the average positioning errors of 6-area partitioning is slightly greater than that of 12-area partitioning. Therefore, it is possible to use 6-area partitioning under acceptable error. 

The earth’s magnetic field has a specific direction and magnitude so the mobile phone must be held by hand at the same angle to ensure sufficient accuracy. This study uses a rotation matrix to convert the original vector coordinates into new coordinates. If the pitch angle is changed, the average positioning error is within 1.4 m, which is about 68% better than the result for the original coordinates. 

The effects from different brands of mobile phones with different magnetometers are also shown in our experiments. The average positioning error using the original coordinates is less than 1.2 m for all applied phones. Thus, using the original coordinates, it is hard to affect the positioning performance in different mobile phones. By using the converted coordinates to ease the same direction constraint between static Earth magnetic field and moving magnetometers in mobile phones, the average positioning error merely increases to 1.96 m. These results show that under the proposed method, different brands of mobile phones give similar indoor positioning results, so the effects of different sensors can be either minimized or eliminated.

## Figures and Tables

**Table 1:** The proposed method summaries between proposed work and related ones.

Applied Methods in Proposed Work	Proposed Work	[18]	[19]	[20]	[21]	[22]
The Earth’s Magnetic Field for Indoor Positioning	Yes	Yes	Yes	Yes	No	Yes
Use Wi-Fi Signal Strength to Perform Indoor Partitioning Technology with KNN	Yes	No	No	Yes	Yes (PDR)	Yes (PDR)
Use the Rotation Matrix to Normalize the Coordinates of the Geomagnetic Field Strength	Yes	No	Yes	No	No	No
Offline and Online System Operation Procedure	Yes	No	Yes	No	No	No
Area Partitioning	Yes	Yes	No	No	No	No

**Table 2:** Magnetometers for mobile phones.

Smartphone Model	Operating System	Magnetometer	Magnetometer Manufacturer
Mi Lite8	Android 10	Ak09918	AKM
Asus Zenfone6	Android 11	Ak0991x	AKM
Google Pixel3	Android 10	LIS2MDL	STMicro
Samsung Galaxy A8+	Android 9	Ak09918	AKM
HTC A9	Android 7	HTC Corp.	HTC Corp.

**Table 3:** Average positioning errors for different numbers of partitioning.

Number of Area	1	4	6	12
Mean Positioning Error	5.47 m	2.18 m	0.93 m	0.57 m

**Table 4:** Average positioning errors without rotation matrix for the EMF [18] and the proposed method.

Number of Areas	4	12
Proposed method	2.18 m	0.57 m
EMF	2.00 m	Not available

**Table 5:** Average positioning errors for converted coordinates.

Number of Areas	1	4	6	12
Average positioning errors	8.73 m	1.56 m	1.49 m	1.36 m

**Table 6:** Average positioning errors for different angles without and with the rotation matrix.

Angle	0°	30°	60°
Mean positioning error of the initial coordinate	0.57 m	3.26 m	3.41 m
Mean positioning error of the conversion coordinate	1.36 m	1.05 m	1.18 m

**Table 7:** Average positioning errors for different brands of mobile phone.

Smartphone Model	Mi Lite8	HTC A9	Asus Zenfone6	Google Pixel3	Samsung Galaxy A8+
Average positioning error	0.57 m	0.86 m	0.41 m	0.83 m	1.15 m

**Table 8:** Average positioning errors for the converted coordinates for different brands of mobile phone.

Smartphone Model	Mi Lite8	HTC A9	Asus Zenfone6	Google Pixel3	Samsung Galaxy A8+
Average positioning error	1.36 m	1.96 m	0.96 m	0.80 m	1.44 m

**Table 9:** The average positioning errors for different distances between reference points.

Distance between Reference Point	1 m	2 m	3 m
Average positioning error of the device related coordinates	0.57 m	1.28 m	1.33 m
Average positioning error of the converted coordinates	1.36 m	1.57 m	1.88 m

**Table 10:** The average positioning error with using only northern database for different distances between reference points.

Distance between Reference Point	1 m	2 m	3 m
Average positioning error of the original coordinates	0.57 m	1.50 m	2.94 m
Average positioning error of the converted coordinates	1.36 m	1.77 m	2.89 m

## Data Availability

Not applicable.

## References

[1] Bahl P., Padmanabhan V.N. (2008). Enhancements to the RADAR User Location and Tracking System.

[2] Liu W., Zhang Y., Deng Z., Zhou H. (2023). Low-Cost Indoor Wireless Fingerprint Location Database Construction Methods: A Review. IEEE Access.

[3] Fazzinga B., Flesca S., Furfaro F., Parisi F. (2016). Exploiting Integrity Constraints for Cleaning Trajectories of RFID-Monitored Objects. ACM Trans. Database Syst..

[4] Wang J., LE J. (2022). Research Progress of RFID Data Cleaning Technology. J. Front. Comput. Sci. Technol..

[5] Baba A.I., Lu H., Pedersen T.B., Xie X. Handling false negatives in indoor RFID data. Proceedings of the 2014 15th IEEE International Conference on Mobile Data Management.

[6] Fazzinga B., Flesca S., Furfaro F., Parisi F. (2020). Interpreting RFID tracking data for simultaneously moving objects: An offline sampling-based approach. Expert Syst. Appl..

[7] Thiagarajan A., Ravindranath L., LaCurts K., Madden S., Balakrishnan H., Toledo S., Eriksson J. VTrack: Accurate, energy-aware road traffic delay estimation using mobile phones. Proceedings of the 7th ACM Conference on Embedded Networked Sensor Systems (SenSys).

[8] Brzozowski B., Kaźmierczak K. Magnetic Field Mapping as a Support for UAV Indoor Navigation System. Proceedings of the 2017 IEEE International Workshop on Metrology for AeroSpace (MetroAeroSpace).

[9] Grisales Campeón J.P., López S., de Jesús Meleán S.R., Moldovan H., Parisi D.R., Fierens P.I. Indoor Positioning Based on RSSI of WiFi Signals: How Accurate Can It Be?. Proceedings of the 2018 IEEE Biennial Congress of Argentina, ARGENCON, Seccion.

[10] Harrington P. (2008). Machine Learning in Action.

[11] Zhao H., Huang B., Jia B. Applying Kriging Interpolation for WiFi Fingerprinting Based Indoor Positioning Systems. Proceedings of the 2016 IEEE WCNC.

[12] Dong Y., Arslan T., Yang Y., Ma Y. A WiFi Fingerprint Augmentation Method for 3-D Crowdsourced Indoor Positioning Systems. Proceedings of the 2022 IEEE IPIN.

[13] Zou H., Chen C.-L., Li M., Yang J., Zhou Y., Xie L., Spanos C.J. (2020). Adversarial Learning-Enabled Automatic WiFi Indoor Radio Map Construction and Adaptation with Mobile Robot. IEEE Internet Things J..

[14] Mendoza-Silva G., Richter P., Torres-Sospedra J., Lohan E., Huerta J. (2018). Long-Term WiFi Fingerprinting Dataset for Research on Robust Indoor Positioning. Data.

[15] Huang H., Lee D.H., Chang K., Li W., Acharya T.D., Lohan E., Huerta J. (2018). Development of Mobile Platform for Indoor Positioning Reference Map Using Geomagnetic Field Data. Comput. Electr. Eng..

[16] Kim H.-S., Seo W., Baek K.-R. (2017). Indoor Positioning System Using Magnetic Field Map Navigation and an Encoder System. Sensors.

[17] Haryanto D.K., Karyono K., Hutagalung S. The Comparison Between Geo magnetism and WiFi for Indoor Positioning System for Public Places. Proceedings of the 2018 IEEE Robionetics.

[18] Yeh S.-C., Hsu W.-H., Lin W.-Y., Wu Y.-F. (2020). Study on an Indoor Positioning System Using Earth’s Magnetic Field. IEEE Trans. Instrum. Meas..

[19] Kang R., Cao L. Smartphone Indoor Positioning System Based on Geomagnetic Field. Proceedings of the 2017 Chinese Automation Congress.

[20] Ning F.-S., Chen Y.-C. (2020). Combining a Modified Particle Filter Method and Indoor Magnetic Fingerprint Map to Assist Pedestrian Dead Reckoning for Indoor Positioning and Navigation. Sensors.

[21] Deng Z.-A., Wang G., Qin D., Na Z., Cui Y., Chen J. (2016). Continuous Indoor Positioning Fusing WiFi, Smartphone Sensors and Land-marks. Sensors.

[22] Li Y., Zhuang Y., Lan H., Zhou Q., Niu X., El-Sheimy N. (2016). A Hybrid WiFi/Magnetic Matching/PDR Approach for Indoor Navigation with Smartphone Sensors. IEEE Commun. Lett..

[23] Lynch K.M., Park F.C. (2017). Modern Robotics: Mechanics.

[24] Kim P., Chae H.G. (2013). Rigid Body Dynamics for Beginners: Euler Angles & Quaternions.

[25] Paul R.P. (1981). Robot Manipulators: Mathematics, Programming, and Control.

[26] Android for Developers: SensorManager. http://developer.android.com/reference/android/hardware/SensorManager.

